# POWERbreathe^®^ Inspiratory Muscle Training in Amyotrophic Lateral Sclerosis

**DOI:** 10.3390/jcm11226655

**Published:** 2022-11-09

**Authors:** Davinia Vicente-Campos, Sandra Sanchez-Jorge, J. L. Chicharro, Ricardo Becerro-de Bengoa-Vallejo, David Rodriguez-Sanz, Arianne R. García, Marie Rivoire, Astrid Benet, Sofía Boubekeur, César Calvo-Lobo

**Affiliations:** 1 Faculty of Health Sciences, Universidad Francisco de Vitoria, Pozuelo de Alarcón, 28223 Madrid, Spain; 2 Grupo FEBIO, Universidad Complutense de Madrid, 28040 Madrid, Spain; 3 Faculty of Nursing, Physical Therapy and Podiatry, Universidad Complutense de Madrid, 28040 Madrid, Spain

**Keywords:** amyotrophic lateral sclerosis, breathing exercises, disability evaluation, heart rate, maximal respiratory pressures, quality of life

## Abstract

Inspiratory muscle training may benefit respiratory function, cardiocirculatory parameters, quality of life and functionality in neuromuscular diseases. This pilot study aimed to demonstrate the POWERbreathe^®^ inspiratory muscle training effects on maximum inspiratory pressure (PI_max_), heart rate (HR) and HR variability, as well as the quality of life impairment and functionality in patients with Amyotrophic Lateral Sclerosis (ALS). A pilot single-blinded, non-randomized controlled clinical trial was carried out. A total of 20T ALS patients were enrolled and divided into experimental (*n* = 10) and control (*n* = 10) groups. The experimental group received POWERbreathe^®^ inspiratory muscle training in conjunction with usual care, and the control group received only usual care for 8 weeks. PI_max_ (measured by POWERbreathe^®^ KH1), HR and HR variability (evaluated by Polar H7), quality of life impairment [measured by the Amyotrophic Lateral Sclerosis Assessment Questionnaire—40 items (ALSAQ-40)] and functionality [assessed by the ALS Functional Rating Scale Revised (ALSFRS-R)] were collected at baseline and after 8 weeks of intervention. We detected statistically significant differences (*p* < 0.05) with an effect size ranging from medium to large (Cohen’s *d* = 0.72–1.37); relative to the control group, the experimental group had an increased PI_max_ (mean difference = 10.80 cm H_2_O; 95% CI = 3.42–18.17) and ALSFRS-R score (mean difference = 5.30 points; 95% CI = −0.03–10.63) and reduced HR (mean difference = −8.80 beats-per-minute; 95% CI = −20.27–2.67) and R-R interval (mean difference = 78.30 ms; 95% CI = 2.89–153.70). POWERbreathe^®^ inspiratory muscle training, in addition to usual care, may improve inspiratory strength and heart rate in patients with ALS. These results encourage larger and longer trials investigating potential clinically relevant benefits of inspiratory muscle training to these patients over the disease course.

## 1. Introduction

Amyotrophic Lateral Sclerosis (ALS) may be defined as a progressive upper and lower motor neuron degenerative condition generating function loss of the motor neurons located in the brain and spinal cord and leading to muscle weakness, wasting, and, ultimately, paralysis [1,2]. In addition, ALS may affect up to 16,000 individuals showing a survival prognosis from 2 to 5 years and needing interdisciplinary management to provide physical and psychological care [1].

Respiratory failure may be the main motor neuron disease symptom contributing to death in ALS patients, with the diaphragm muscle being considered the most important for those cases progressing to respiratory muscle weakness and paralysis [3,4,5]. Among neuromuscular diseases, inspiratory muscle training has been proposed as a novel intervention in conjunction with usual care, which may potentially strengthen the diaphragm muscle and minimize respiratory dysfunction in ALS patients [6,7].

During the last decades, inspiratory muscle training protocols have been applied by exercises and diaphragm reeducation for at least 8 weeks, showing pulmonary function improvements as well as even an increase in survival in early-affected patients with ALS [6,7,8]. Nevertheless, there is a lack of knowledge about inspiratory muscle training by the POWERbreathe^®^ device (POWERbreathe International Ltd., Southam, Warwickshire, UK) in patients who suffer specifically from ALS, and this device has recently shown benefits on pulmonary function as an adjunct intervention to usual care in neuromuscular diseases [9].

According to the reported benefits of inspiratory muscle training on respiratory function, cardiocirculatory parameters, quality of life and functionality in neuromuscular diseases [6,7,8,9], we hypothesized that the POWERbreathe^®^ device could provide these benefits to the usual care received in patients with ALS. Thus, the primary aim was to demonstrate the POWERbreathe^®^ device’s effects on maximum inspiratory pressure (PImax) to strengthen the diaphragm muscle and minimize muscle weakness and wasting in patients who suffered from ALS. In addition, the secondary purposes were to determine the effects of inspiratory muscle training by this tool on heart rate and variability as well as ALS patients’ quality of life and functionality.

## 2. Materials and Methods

This study was a pilot single-blinded (outcome assessor), non-randomized controlled clinical trial carried out from May to November 2021, following the Consolidated Standards of Reporting Trials (CONSORT) criteria and checklist [10].

### 2.1. Participants

A total of 20 patients (9 men, 11 women), aged from 38 to 70 years (mean age ± standard deviation (SD) of 49.6 ± 8.6 years), with bulbar or spinal ALS onset, were recruited by a consecutive sampling method by an advertisement in the newsletter of the ADELA patients’ association (Spanish Association of Amyotrophic Lateral Sclerosis Patients, Madrid, Spain). Participants were divided into experimental (*n* = 10) and control (*n* = 10) groups by a non-randomization procedure to achieve similar age and sex paired-matched groups to avoid baseline between-groups statistical differences due to the low sample size.

A sample size calculation was carried out using the G*Power software (version 3.1.9.2, G*Power©; Universität Kiel, Germany), guided by the t-family tests by the mean difference between two independent groups of a prior study [11]. According to the primary outcome measurement of maximum inspiration pressure (PImax), a prior study carried out by Smeltzer et al. [12,13] in ALS patients divided into experimental and control groups, showed a PImax mean ± SD difference (post-pre) after the intervention of 35.50 ± 28.00 mmHg and 4.90 ± 14.80 mmHg, respectively, providing a large effect size (*d* = 1.36) [14]. Considering a two-tailed hypothesis, an α value of 0.05 and a power of 0.80 in addition to the described large effect size, a total sample size of 20 patients, 10 ALS patients for each group, was necessary to achieve an actual power of 0.82.

Inclusion criteria comprised patients with: (1) less than 1.5 years from the actual medical diagnosis; [2] (2) a PImax greater than 30 cm H_2_O [6,7,8]; (3) age ranges between 18 to 65 years; and (3) with or without Riluzole treatment [15]. 

Exclusion criteria included patients with: (1) Reisber’s Global Deterioration Scale (GDS) score for cognitive impairment greater than 2 points [16]; (2) prior initiated respiratory muscle strength training; (3) ventilatory support through a tracheostomy or noninvasive ventilation for more than 14 h per day [6]; (4). prior diagnosis of co-existing respiratory or neurological disease; (5) unstable condition in the preceding 3 years in the medical record and (6) PImax measurements contraindications, such as unstable angina, recent acute myocardial infarction (previous 4 weeks), myocarditis, uncontrolled systemic hypertension, recent pneumothorax, postoperative pulmonary biopsy in the last week, abdominal or genitourinary surgery in the last 6 months and urinary incontinence [6].

The present research was approved by the ethics review board (approval code: 35/2021) from the Francisco de Vitoria University (Madrid, Spain) and registered at ClinicalTrials.gov (Identifier: NCT04889248). Furthermore, all ALS patients provided their signed informed consent forms to participate in this study. All ethical requirements, including Helsinki Declaration and Human Rights for biomedical investigation, were respected [17,18].

### 2.2. Interventions

Participants were non-randomly assigned to an experimental (*n* = 10) group (receiving POWERbreathe^®^ inspiratory muscle training in conjunction with usual care) and a control (*n* = 10) group (receiving only usual care). A blinding procedure was applied to the outcome assessor, who did not know the intervention allocation for each patient.

The experimental group received inspiratory muscle training in addition to usual care by the POWERbreathe^®^ device (POWERbreathe International Ltd., UK). This inspiratory muscle training presented 11 resistance levels ranging from 23 cm H_2_O minimum to 186 cm H_2_O, allowing incremental resistance progression, and was applied for 8 weeks according to a prior published protocol applied in patients with neuromuscular disease [19]. This protocol consisted of 30 inspirations per day, divided into 15 repetitions in the morning and 15 repetitions in the evening, 5 days per week, with resting at the weekend for 8 weeks. The training resistance was applied following an incremental protocol according to the PImax measured at the first visit. During the first week, the training resistance was set to 30% of PImax. The training resistance was increased to 40% of PImax during the second and third weeks, 50% of PImax during the fourth and fifth weeks and 60% of PImax during the sixth, seventh and eighth weeks [19]. The inspiratory muscle training was applied domiciliary but supervised to avoid a loss of follow-up due to daily phone calls, emails and videoconferences to ensure patients’ adherence and domiciliary protocol compliance. In addition, the inspiratory muscle training workload was weekly adjusted by face-to-face domiciliary consultations. Daily phone calls and/or videoconferences were used to check compliance with daily training.

**Figure 1:**
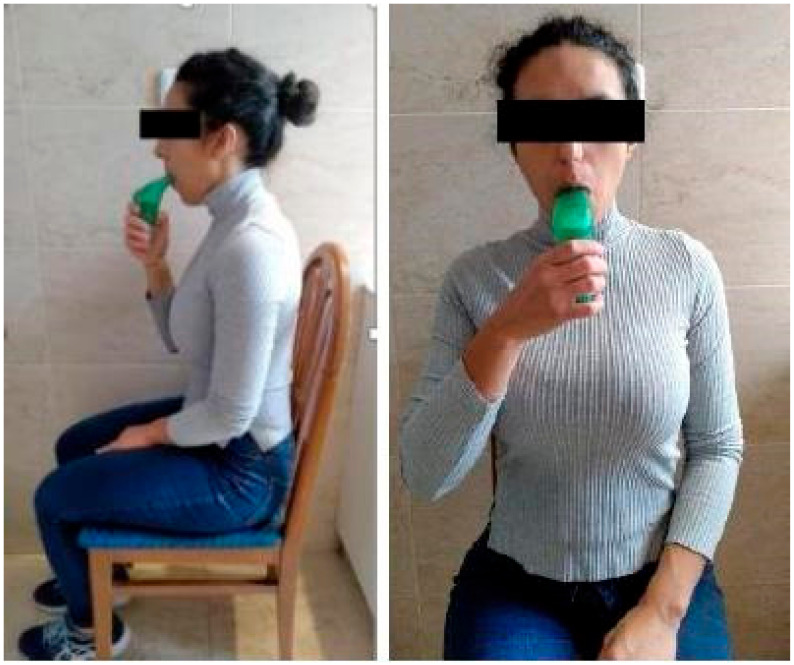
Domiciliary inspiratory muscle training with the POWERbreathe^®^ device.

Patients were asked to perform full and deep breaths but not at maximum power. Thus, the concept of maximum power and the modified Borg scale [20] were explained. The modified Borg scale evaluates effort from 0, which corresponds to “nothing at all”, to 10, which corresponds to “extremely strong (almost max)”. It is a validated tool when someone wants to describe how the subjective intensity varies with the physical intensity. They were asked to train at a level from 6 to 7 points on this scale, avoiding counterproductive fatigue, starting from a residual volume without exceeding 2 min of resting between each inspiration [21]. The prescribed training sessions were performed in a seated and relaxed position (Figure 1), before eating or 2 h later, with a minimum interval of 3 h between each session and weekly supervised to adjust the workload for inspiratory muscle training. Both experimental and control groups received usual care, with or without Riluzole treatment [15], according to the prescribed care by their medical specialists [22,23,24].

### 2.3. Outcome Measures

Descriptive data were registered at baseline, including sex (male or female), site of symptom onset (bulbar or spinal ALS), age (years), weight (kg) and height (m). Primary (PImax) and secondary outcomes (heart rate and variability and ALS patients’ quality of life and function) were measured at baseline and after 8 weeks coinciding with the final inspiratory muscle training.

Once the inclusion process was completed, two researchers went on the first visit to each of the subjects’ homes to explain the study and obtain the baseline measurements. These measurements were taken in the following order to avoid interferences: heart rate and heart variability, questionnaires and PImax. Subsequently, on a second day, familiarization with the POWERBreathe^®^ device was carried out by one of the expert researchers in respiratory muscle training so that the patients could train autonomously every day at home.

#### 2.3.1. Maximum Inspiration Pressure

The strength of the inspiratory muscles, mainly the diaphragm, was measured in cm H_2_O by the POWERbreathe^®^ KH1 device (POWERbreathe International Ltd., UK) to determine the PImax measurements by a piezoelectric pressure transducer, with an accuracy of 0.367 cm H_2_O and a pressure range of ±147 cm H_2_O. This tool presented a valvular device with an opening for a small leak to prevent glottis closure during the PImax maneuver. In addition, a connection filter was placed between the mouthpiece and the measuring equipment, according to international recommendations [25]. We opted to use a ‘diving type’ mouthpiece since these are commonly used and confer greater patient comfort and improved coordination to perform the maneuvers. The equipment was calibrated in each measurement according to the manufacturer’s considerations. Measurements were carried out in a sitting position. The instructions for inserting the mouthpiece were that the tongue should not be inserted or bitten, the seal should be maintained without leakage with the lips around the mouthpiece, and nasal forceps were used to avoid air leakage through the nostrils during the measurement of PImax [26].

This procedure was repeated three times in each patient, with a minimum exhalation duration of 1.5 s, to obtain the average measurement of 1 s. Patients were first asked to perform a cycle of three slow and normal breaths, then asked to exhale gently until reaching residual lung volume and finally to perform a rapid and maximal inspiration accompanied by verbal encouragement from the examiner to perform the test as vigorously as possible. Among the three repetitions, a rest of at least 60 s was performed between attempts. Values equal to or higher than 30 cm H_2_O were considered acceptable. Of these three values, the highest value was registered for the study [26].

#### 2.3.2. Heart Rate and Variability

Heart rate (HR) and HR variability (HRV) were measured using a Polar H7 device connected to the Elite HRV application, used as a noninvasive biomarker and validated tool [27,28]. Values were recorded in beats per minute (bpm) for HR and milliseconds for HRV as a biomarker of the function of the autonomic nervous system (ANS) expressed through the sympathetic and parasympathetic modulation of HR, indicating higher values for a better health state [29]. Patients were asked to sit in a comfortable position with their arms relaxed on the armrests for 10 min; the Polar H7 elastic band was adjusted around the chest, just below the pectoral muscles in men and at the xiphoid appendix level in women [29]. 

First, the average R-R interval duration of measurement (R-R INTERVAL) corresponded to the time between each heartbeat expressed in milliseconds. A high R-R value was determined by fewer beats per time unit and, therefore, a lower resting HR. Second, the root means square of the successive differences (rMSSD) was expressed in milliseconds and defined as the expression of the activity of the ANS, mainly through the vagus nerve (cranial nerve X) that linked the brain to the heart and, therefore, provided accurate information about the relationship between HR and the patient’s nervous system health. Lastly, the standard deviation of the Normal-to-Normal Bits (SDNN) recorded the fluctuations of HR, indicating greater fluctuation for a better patient health state showing the patient’s responsiveness in the transition from a stressful situation to a resting state [30].

#### 2.3.3. Quality of Life Impairment

ALS patients’ quality of life was measured by the Amyotrophic Lateral Sclerosis Assessment Questionnaire—40 items (ALSAQ-40). This tool measured the disease involvement level and progression by 40 items grouped into five representative quality of life domains, such as physical mobility, activities of daily living, eating and drinking, communication and emotional function. The ALSAQ-40 was validated, scoring from 0 to 160 points, 0 points being considered the best prognosis and 160 points considered the worst score indicating greater quality of life impairment [31].

#### 2.3.4. Functionality

ALS patients’ functionality was measured by the ALS Functional Rating Scale Revised (ALSFRS-R), developed specifically for the ALS population in clinical trials and based on physical function scores for activities of daily living. This tool presented 12 items related to the physical functional aspect divided into four parts: bulbar, fine motor, gross motor and respiratory. This questionnaire was Spanish validated and scored from 0 points indicating worst functionality to 52 points indicating the best functionality. Therefore, decreasing the ALSFRS-R score may support a worse disease prognosis and survival [32,33]. The ALSFRS-R was applied by a blinder evaluator to derive the total score and the bulbar function subscore.

### 2.4. Statistical Analysis

Statistical Package for Social Sciences (SPSS) 24.0 version (IBM; Armonk, NY, USA; IBM–Corp) was used to carry out statistical analyses by α of 0.05 and statistically significant of *p* < 0.05 for a 95% confidence interval (CI).

Regarding quantitative data, the Shapiro–Wilk test was applied to determine normality distribution. Next, the Shapiro–Wilk test demonstrated parametric distribution if *p* ≥ 0.05. In addition, the Shapiro–Wilk test demonstrated non-parametric distribution if *p* < 0.05. All data were described as mean ± standard deviation (SD) and mean differences completed with their lower and upper limits for 95% CI, detailing the t statistic for parametric distribution and *U* statistic for non-parametric distribution.

According to between-group comparisons, *p*-values of the Student’s *t*-test for independent samples were used for parametric data according to Levene’s test for equality of variances. Furthermore, *p*-values of the Mann–Whitney *U* test for independent samples were used for non-parametric data. In addition, sex distribution was compared by the Fisher exact test. For outcome measurement differences after interventions, effect size was determined by the Cohen’s d and interpreted as very small effect size (*d* < 0.20), small effect size (*d* = 0.20–0.49), medium effect size (*d* = 0.50–0.79) and large effect size (*d* > 0.8) [34].

## 3. Results

**Figure 2:**
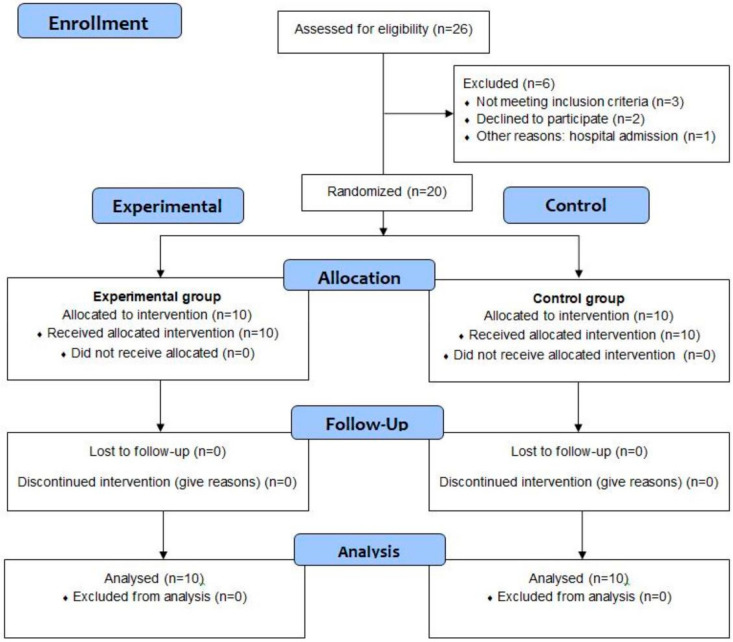
Flow diagram.

Of 26 patients diagnosed with ALS (bulbar or spinal), 6 patients were excluded: 3 did not meet inclusion criteria, 2 declined to participate and 1 received hospital admission (Figure 2). Thus, 20 patients were divided into the experimental group (*n* = 10 patients received POWERbreathe^®^ inspiratory muscle training plus usual care) and the control group (*n* = 10 patients received only usual care). Baseline data did not show any statistically significant differences (*p* > 0.05) between both experimental and control groups for descriptive data or outcome measurements (Table 1). Indeed, bulbar function subscores of the ALSFRS-R did not show significant differences (*p* > 0.05) between both groups at baseline. In addition, the Fisher exact test did not show any statistically significant differences (*p* = 1.00) for sex distribution and site of symptom onset. There was only one patient with bulbar ALS in the experimental group; all other patients in the experimental and control groups presented spinal ALS.

Regarding between-groups comparisons of the outcome measurements after interventions, there were statistically significant differences (*p* < 0.05) with an effect size ranging from medium to large (Cohen’s *d* = 0.72–1.37) showing that the experimental group had an increased PImax (mean difference = 10.80 cm H_2_O; 95% CI = 3.42–18.17; *U* = 5.500) and ALSFRS-R score (mean difference = 5.30 points; 95% CI = −0.03–10.63; *U* = 23.500), as well as a reduced HR (mean difference = −8.80 bpm; 95% CI = (−20.27–2.67; *U* = 79.500) and R-R interval (mean difference = 78.30 ms; 95% CI = 2.89–153.70; *U* = 19.000) relative to the control group (Table 2). The rest of the outcome measurement differences comparisons did not show any statistically significant difference (*p* > 0.05), with an effect size ranging from very small to medium (Cohen’s *d* = 0.15–0.90) between both groups after interventions. Concretely, bulbar function subscores did not show any significant difference (*p* > 0.05), with a very small effect size (Cohen’s *d* = 0.00–0.15), between both groups after intervention. 

## 4. Discussion

To the authors’ knowledge, this study may be considered a novel pilot clinical trial analyzing the effects of 8-week domiciliary and supervised inspiratory muscle training by the POWERbreathe^®^ in conjunction with usual care in patients who suffer from ALS. 

Our results show a significant increase (*p* < 0.05) [with an effect size ranging from medium to large (Cohen’s *d* = 0.72–1.37)] for the PImax in the experimental group but not in the control group (in which not only the strength of the inspiratory muscle was not improved, but there was a tendency for it to decrease). In these patients, in whom spontaneous breathing is affected by a loss of motor neurons of the spinal cord anterior horn responsible for innervation of the diaphragm, any intervention that could delay this loss of function of the diaphragm and the rest of respiratory muscles is of vital importance. Our results support other studies showing the positive effect of respiratory muscle training on respiratory muscle strength in ALS patients [6,8,35,36] or other neurological disorders, such as multiple sclerosis [37,38]. Cheah et al. [6] show an increase in respiratory muscle strength after 12 weeks of training and point out that measures of respiratory function suggest that IMT may have partially ameliorated the restrictive defect that develops in ALS patients. Subsequently, Pinto et al. [35] studied the effect of 8 months of IMT in ALS patients and, although their results did not suggest that inspiratory exercise can defer respiratory function decline in early affected ALS patients with normal respiratory function, their observations suggest a transitory mild benefit, which is more evident on clinical evaluations and in tests evaluating respiratory fatigue, such as maximum voluntary ventilation. Perhaps, 8 months of intervention is too long for a disease with such a rapid evolution. 

In addition, in another study, Pinto et al. [8] also measured the value of phrenic nerve response amplitude, as it is an independent prognostic factor for survival in ALS patients and observed an improvement with IMT performed through an inspiratory resistive device, Threshold^®^ IMT, from Respironics. 

On the other hand, Nardin 2008 [35] completed a study of diaphragmatic training in 10 ALS/MND patients with respiratory impairment, with the aim to strengthen inspiratory muscles. Instead of using an IMT device, patients in that study engaged in diaphragmatic training (DT), a breathing technique that required conscious awareness of diaphragmatic contractions. They found that the subjects who performed the DT, assessed by respiratory magnetometry, tended to slow their rate of decline in FVC compared with those who did not perform the technique. Although this was not statistically significant, the trend was suggestive.

Regardless of the small sample sizes of the studies to date, their results together with ours would tend to support the hypothesis that despite an environment of ongoing denervation, the inspiratory muscles of ALS patients can respond favorably to a strength training program. 

In addition to classical motor impairments, dysfunction of the autonomic nervous system (ANS) may occur during ALS regardless of the disease stage [39,40,41,42,43,44,45], and recent evidence indicates that such dysfunction may worsen the quality of life and influence survival, particularly in the advanced stages of the disease [41,43,44,45]. In this sense, HRV is a widely-used physiological marker of sympathovagal balance that can be reliably measured in real-world settings [46]. Specifically, higher resting heart rates and lower heart rate variability were found in ALS patients compared to healthy controls [39,47]. To our knowledge, this is the first study to investigate the effects of inspiratory muscle training on ANS through the HRV and the resting heart rate in these patients. We detected a reduction of resting HR in the trained group, suggesting a lower resting heart function, but the other ANS parameters did not show statistically significant differences [30]. The short duration of the training protocol (8 weeks) and the small sample size might account for us not observing changes in these parameters.

Despite a moderate effect size between both groups, the improvement of the quality of life impairment did not reach significant differences, and this fact may be due to the duration of the inspiratory muscle training—only 8 weeks versus 12 weeks proposed in neuromuscular diseases [6,35]—as well as different inspiratory muscle training devices different from POWERbreathe^®^, such as threshold devices [6,26]. 

Despite the low sample size of our study, prior research studies applying inspiratory muscle training in ALS patients showed a smaller sample size than the present study [6,9]. Other authors have proposed that the lack of participation and/or follow-up of this type of patient is because of disability or difficulty of transport to and from the hospital [6,8,35,36]. In our study, the inspiratory muscle training was applied domiciliary but supervised to avoid a loss to follow-up due to daily phone calls and videoconferences to ensure patients’ adherence and domiciliary protocol compliance. None of the patients in our study showed any discomfort or difficulties when performing the training, as they commented daily on the phone calls or videoconferences with the researchers. Thus, respiratory muscle training seems to be a safe and easy-to-use tool in ALS patients, although larger sample studies related to this fact are needed in this area. Deaths, hospital admissions, complications and worsening prognoses may be common in patients who suffer from ALS, making it challenging to find enough patients for a clinical study [1].

## 5. Limitations

First, the low sample size and the 8-week inspiratory muscle training protocol should be increased in future studies. Second, we performed a non-randomized clinical trial to avoid sex and age differences according to the low sample size, but future studies should carry out randomized clinical trials to avoid bias. In addition, our control group received only usual care without inspiratory muscle training, and future clinical trials should be controlled using POWERbreathe^®^ without workload [36].

In our sample, there was only one patient with bulbar onset ALS in the experimental group, and this patient was in a very early stage of the disease. For future studies, it would be necessary to include more bulbar onset patients, even comparing the two types of onset symptoms (bulbar and spinal). Indeed, it could be very interesting to include the bulbar function level of the patients at the time they train.

Lastly, we have not included the hours per day with NIV, which could be a confounder for the results; although we did include it as an exclusion criterion, the patients could not use noninvasive ventilation for more than 14 h per day. It would be very interesting to also include vital capacity and disease duration to complete the description of the sample and strengthen the results.

## 6. Conclusions

POWERbreathe^®^ inspiratory muscle training (in addition to usual care) may improve inspiratory muscle strength and resting heart rate in patients with ALS. These results encourage larger and longer trials investigating potential clinically relevant benefits of inspiratory muscle training in these patients over the disease course.

## Figures and Tables

**Table 1:** Baseline data between experimental and control groups.

Baseline Data	Total Sample (*n* = 20) Mean ± SD (95% CI)	Experimental (*n* = 10) Mean ± SD (95% CI)	Control (*n* = 10) Mean ± SD (95% CI)	Mean Difference (95% CI)	Statistics	*p*-Value
Age (years)	49.6 ± 8.6 (45.6–53.6)	49.1 ± 10.9 (41.3–56.9)	50.1 ± 6.2 (45.7–54.6)	−1.00 (−9.3–7.3)	*t* = −0.253	0.803 *
Weight (kg)	67.9 ± 10.4 (63.1–72.8)	68.5 ±14.4 (58.2–78.8)	67.4 ± 4.3 (64.3–70.5)	1.1 (−9.4–11.6)	*t* = 0.231	0.822 *
Height (m)	1.7 ± 0.1 (1.6–1.7)	1.6 ± 0.1 (1.6–1.7)	1.7 ± 0.1 (1.6–1.7)	−0.03 (−0.09–0.03)	*t* = −0.990	0.336 *
PI_max_ (cm H_2_O)	50.8 ± 16.1 (43.3–58.3)	50.3 ± 19.9 (36.0–64.6)	51.3 ± 12.0 (42.7–59.9)	−1.0 (−16.5–14.5)	*t* = −0.136	0.894 *
HR (bpm)	90.6 ± 12.5 (84.8–96.4)	93.0 ± 14.9 (82.3–103.7)	88.2 ± 9.6 (81.3–91.1)	4.8 (−6.9–16.6)	*U* = 41.500	0.529 ^†^
HRV (ms)	44.4 ± 9.1 (40.1–48.7)	44.1 ± 11.4 (35.9–52.2)	44.7 ± 6.8 (39.8–49.6)	−0.6 (−9.4–8.2)	*U =* 57.500	0.579 ^†^
R-R interval (ms)	658.3 ± 75.8 (622.9–693.8)	657.7 ± 90.8 (592.8–722.6)	659.00 ± 62.4 (614.4–703.6)	−1.3 (−74.5–71.9)	*t* = −0.037	0.971 *
rMSSD (ms)	23.4 ± 28.5 (10.0–36.7)	22.9 ± 30.9 (0.8–45.1)	23.8 ± 27.4 (4.2–43.5)	−0.9 (−28.4–26.6)	*U* = 55.500	0.684 ^†^
SDNN (ms)	47.7 ± 31.3 (33.0–62.4)	46.1 ± 33.3 (22.3–69.9)	49.3 ± 31.0 (27.1–71.5)	−3.2 (−33.4–27.0)	*U* = 54.000	0.796 ^†^
ALSAQ-40 (scores)	71.1 ± 30.7 (56.7–85.4)	73.8 ± 33.6 (49.8–97.8)	68.3 ± 29.1 (47.4–89.1)	5.5 (−24.0–35.0)	*t* = 0.391	0.700 *
ALSFRS-R (total score)	34.4 ± 7.8 (31.1–37.7)	33.5 ± 7.5 (28.1–38.9)	35.3 ± 6.8 (30.4–40.2)	−1.8 (−8.5–4.9)	*U =* 61.500	0.393 ^†^
ALSFRS-R bulbar function (subscores)	9.9 ± 1.5 (9.1–10.6)	9.7 ± 1.7 (8.4–10.9)	10.1 ± 1.2 (9.1–11.0)	−0.4 (−1.8–1.0)	*U =* 53.500	0.796 ^†^
ALSFRS-R language (subscores)	3.3 ± 0.4 (3.1–3.5)	3.3 ± 0.4 (2.9–3.6)	3.4 ± 0.5 (3.0–3.7)	−0.1 (−0.5–0.3)	*U =* 55.000	0.739 ^†^
ALSFRS-R salivation (subscores)	3.2 ± 0.6 (2.9–3.5)	3.2 ± 0.7 (2.6–3.7)	3.3 ± 0.4 (2.9–3.6)	−0.1 (−0.7–0.5)	*U* = 52.000	0.912 ^†^
ALSFRS-R swallowing (subscores)	3.0 ± 0.9 (2.5–3.4)	3.0 ± 1.1 (2.1–3.8)	3.0 ± 0.6 (2.5–3.4)	0.0 (−0.9–0.9)	*U =* 44.000	0.684 ^†^

Abbreviations: ALSAQ-40, Amyotrophic Lateral Sclerosis Assessment Questionnaire—40 items; ALSFRS-R, ALS Functional Rating Scale Revised; HR, heart rate expressed in beats per minute (bpm); HRV, heart rate variability expressed in milliseconds (ms); PI_max_, Maximum Inspiration Pressure expressed in centimeters of H_2_O (cm H_2_O); R-R interval, time between each heartbeat expressed in milliseconds (ms); rMSSD, root means square of the successive differences expressed in milliseconds (ms); SDNN, standard deviation of the Normal-to-Normal Bits expressed in milliseconds (ms). * Student’s *t*-test for independent samples used, including *t*-statistic for parametric distribution; ^†^ Mann–Whitney *U* test applied including *U* statistic for parametric distribution. For all analyses, *p* < 0.05 (for a confidence interval of 95%) was considered statistically significant.

**Table 2:** Comparison of outcome measurement differences after experimental (POWERbreathe^®^ inspiratory muscle training plus usual care) and control (only usual care) interventions.

Outcome Differences after Interventions	Experimental (*n* = 10) Mean ± SD (95% CI)	Control (*n* = 10) Mean ± SD (95% CI)	Mean Difference (95% CI)	Statistics	*p*-Value	Effect Size (Cohen *d*)
PI_max_ (cm H_2_O)	5.6 ± 9.8 (−1.4–12.6)	−5.2 ± 5.2 (−8.9–−1.5)	10.8 (3.4–18.2)	*U* = 5.500	**<0.001** ^†^	*d* = 1.37
HR (bpm)	−6.8 ± 17.1 (−19.1–5.4)	2.0 ± 2.3 (0.4–3.6)	−8.8 (−20.3–2.7)	*U* = 79.500	**0.023** ^†^	*d* = 0.72
HRV (ms)	5.1 ± 23.9 (−12.0–22.2)	−3.5 ± 3.2 (−5.8–−1.2)	8.6 (−7.4–24.6)	*U =* 30.500	0.143 ^†^	*d =* 0.50
R-R interval (ms)	44.3 ± 105.1 (−30.9–119.5)	−34.0 ± 42.6 (−64.5–−3.5)	78.3 (2.9–153.7)	*U =* 19.000	**0.019** ^†^	*d =* 0.97
rMSSD (ms)	1.7 ± 38.9 (−26.1–29.6)	−2.6 ± 3.5 (−5.1–−0.1)	4.31 (−21.7–30.3)	*U* = 30.000	0.143 ^†^	*d* = 0.15
SDNN (ms)	0.1 ± 42.4 (−30.3–30.4)	−5.5 ± 9.6 (−12.3–1.4)	5.53 (−23.4–34.4)	*U* = 44.000	0.684 ^†^	*d* = 0.17
ALSAQ-40 (scores)	5.2 ± 6.9 (0.2–10.2)	20.9 ± 23.5 (4.0–37.7)	−15.7 (−32.9–1.5)	*t* = −2.022	0.069 *	*d* = 0.90
ALSFRS-R (total score)	−4.3 ± 3.2 (−6.6–−1.9)	−9.6 ± 7.1 (−14.6–−4.5)	5.3 (−0.03–10.6)	*U* = 23.500	**0.043** †	*d* = 0.94
ALSFRS-R bulbar function (subscores)	−0.8 ± 1.6 (−2.0–0.4)	−1.0 ± 1.6 (−2.1–0.1)	0.2 (−1.3–1.7)	*U* = 42.000	0.579 ^†^	*d* = 0.12
ALSFRS-R language (subscores)	−0.1 ± 0.5 (−0.5–0.3)	0.0 ± 0.4 (−0.3–0.3)	−0.1 (−0.5–0.3)	*U* = 54.500	0.739 ^†^	*d* = 0.09
ALSFRS-R salivation (subscores)	−0.3 ± 0.6 (−0.7–0.1)	−0.2 ± 0.7 (−0.7–0.3)	−0.1 (−0.7–0.5)	*U* = 57.000	0.631 ^†^	*d* = 0.15
ALSFRS-R swallowing (subscores)	0.0 ± 0.6 (−0.4–0.4)	0.0 ± 0.6 (−0.4–0.4)	0.0 (−0.6–0.6)	*U* = 50.000	1.000 ^†^	*d* = 0.00

Abbreviations: ALSAQ-40, Amyotrophic Lateral Sclerosis Assessment Questionnaire—40 items; ALSFRS-R, ALS Functional Rating Scale Revised; HR, heart rate expressed in beats per minute (bpm); HRV, heart rate variability expressed in milliseconds (ms); PI_max_, Maximum Inspiration Pressure expressed in centimeters of H_2_O (cm H_2_O); R-R interval, time between each heartbeat expressed in milliseconds (ms); rMSSD, root means square of the successive differences expressed in milliseconds (ms); SDNN, standard deviation of the Normal-to-Normal Bits expressed in milliseconds (ms). * Student’s *t*-test for independent samples used, including *t*-statistic for parametric distribution; ^†^ Mann–Whitney *U* test applied including *U* statistic for parametric distribution. For all analyses, *p* < 0.05 (for a confidence interval of 95%) was considered statistically significant (**bold**).

## Data Availability

Raw data are available upon corresponding author request.
